# Biology of the Chalcid Wasp, *Megastimus wachtli*, and Its Relationship to Colonization of Cypress Seeds by the Tortricid Moth, *Pseudococcyx tessulatana*, in Algeria

**DOI:** 10.1673/031.006.4801

**Published:** 2006-12-31

**Authors:** K. Bouaziz, A. Roques

**Affiliations:** ^1^ EA 2106 Biomolécules et Biotechnologies végétales, UFR Sciences Pharmaceutiques, 31 avenue Monge 37200 Tours.; ^2^ Institut National de la Recherche Agronomique, INRA-CRF Zoologie Forestière, Ardon, F-45160 Olivet, France

**Keywords:** *Cupressus simpervirens*, *Cupressus arizonica arizonica*, *Cupressus arizonica glabra*, *Nanodiscus transversus*, *Brachyacma oxycedrella*, *Orsillus maculatus*, *Orsillus depressus*, *Caruslaspis minima*, cone, pest insect

## Abstract

The biology of *Megastimus wachtli* Seitner (Hymenoptera: Torymidae) was found to be similar to other species of *Megastigmus.* During the period of flight that lasted six weeks from the beginning of September to mid-October, *M*. *wachtli* laid eggs in cypress (*Cupressus sempervirens* L., Pinales: Cupressaceae) cones and showed preferences for oviposition on particular sites on cones. *M. wachtli* has a high potential for colonization because it has evolutionary advantages due to its developmental possibilities including its capacity for parthenogenesis, its fecundity and longevity. It generally did not attack cones colonized by the torticid moth, *Pseudococcyx tessulatana* (Lepidoptera: Tortricidae). The competition between these species for use of cypress cones suggests that they use different strategies for different species of cypress. The number of insects that could develop relative to the number of cones available also varies between species of cypress.

## Résumé

L'étude de la biologie de *Megastimuswachtli* (Hymenoptera: Torymidae) nous a permis de démontrer que la femelle de *M*. *wachtli* n'est pas différente des espèces voisines de *Megastigmus.* La durée de vole de *M*. *wachtli* s'étale sur 6 semaines du début septembre à la mi-octobre et varie suivant les conditions climatiques. L'espèce *M*. *wachtli* présente des potentialités d'attaq ues très élevées car elle a des avantages évolutifs qui lui permettent de se maintenir et d'avoir une grande diversité du fait ses possibilités de développement. On peut révéler sa longévité élevée, sa fécondité, sa capacité de parthénogenèse (arrhénotoque). Les attaques de *Pseudococcyx tessulatana* (Lepidoptera: Tortricidae) et du chalcidien semblent à avoir tendance à mutuellement s'exclure. Ce qui suggère l'existence de composés anti-oviposition. On peut suggérer, en ce qui concerne la compétition inter et intra spécifique dans l'utilisation de la ressource cône même si les indices utilisés (nombre et position des trous de sorties) donnent qu'une vision imparfaite des processus d'attaques, qu'il existe des différences de stratégie dans l'occupation de l'espace selon les espèces et selon l'abondance des insectes relativement à celle de leur hôte.

## Introduction

The green cypress (*Cupressus sempervirens* L., Pinales: Cupressaceae) is of great interest for ornamental, reforestation and windbreak use in the entire Mediterranean basin. It is well adaptated to the various Mediterranean conditions and resists long dry periods as well as cold extremes. The entomological fauna that exploits Cupressaceae seeds is well known. Roques and Battisti (1999) mention nine insects and acarina species found in cypress seeds and cones in natural forests, and in plantations, seedbeds, seed orchards or urban trees of the Mediterranean area. These authors showed how insects specialized in seed exploitation. The seed chalcid, *Megastigmus wachtli* Seitner (Hymenoptera: Torymidae), and seed bugs, *Orsillus* (Dallas) (Heteroptera: Lygaeidae), limit seed yields, particularly in seed orchards. A total of 21 *Megastigmus* seed chalcid species are recognized in Europe, North Africa and Asia Minor (Roques and Skrzypczynska 2003). Battisti et al. (2003) affirmed that more than 60% of cypress cones were colonized either by a seed chalcid, *M. wachtli*, or by the seed bug, *Orsillus maculatus* (Fieber). A survey of the oviposition behavior of *Orsillus depressus* carried out in Algeria suggested that this seed bug lays eggs in emergence holes excavated through the cone scale by a seed chalcid, *M*. *wachtli* (Bouaziz 2003). Similarly, a survey of the oviposition behavior of *O. maculatus* carried out in France and Italy suggested that this seed bug lays eggs preferentially in the emergence holes of *M*. *wachtli* (Rouault 2002). In addition, the relationship between the exotic pathogenic fungus *Seiridium cardinale* (Wagener) Sutton and Gibson and species of the genus *Orsillus* on *C*. *sempervirens* was shown to be essentially based on the availability of *M*. *wachtli* oviposition sites (Battisti et al. 1997, Battisti et al. 1999; Battisti et al. 2000; Bouaziz 2003; Rouault et al. 2000, Rouault 2002).

The tortricid *Pseudococcyx tessulatana* Staudinger (Lepidoptera: Tortricidae) and the seed chalcid *M*. *wachtli* also found in Algeria (Bouaziz 1993; Bouaziz and Chakali 1997) seemed to cause significant damage (Guido et al. 1995; Roques and Raimbault 1986; Zocchi 1963). However, their exact distribution, and their biology, remains unstudied in Algeria.

This article aims to establish: (i) the identity of entomofauna attacking seed cones in the Baïnem cypress arboreta. For this purpose seed cones of *C*. *sempervirens*, were monitored over its entire development, from flower appearance to seed maturity; (ii) the biological parameters of the development of *M*. *wachtli* on cypress; (iii) the respective impact of *M*. *wachtli* and *P*. *tessulatana* on seed cones of *Cupressus* spp. (iv) the relationships among individuals of the same insects exploiting seed cones and between species of insects on the cones; and (v) the distribution of the *M*. *wachtli* and the tortricid emergence holes on the cones.

## Materials and Methods

### Study sites

The study was carried out in the Baïnem arboretum, located at 15 km west of Algiers. This arboretum was set up in 1956, in the center of the Baïnem forest, where it occupied 50 ha of the 500 ha of this forest (longitude 2° 57′ 20″ to 2° 57′ 57″ and latitude 36° 47′ 16″ to 36° 48′ 06″). The soil was clay on schist with many coarse elements. The altitude varies from 195 to 215 meters, with an average of 200 meters. The climate is of the Mediterranean type, sub-wet with hot summers characterized by dry and a hot period extending from May to September and a rainy period from October to May. Three cypress species were examined: *C*. *sempervirens*, *C*. *arizonica arizonica*, and *C*. *arizonica glabra.*

The *C*. *arizonica arizonica* stand was started in 1959 on a declivity from 20 to 30 % with geographical coordinates: 2° 57′ 46″ of longitude and 36° 48′ of latitude, with 132 trees and the general exposure is Northwest.

The *C. arizonica glabra* stand was started on March 31 1959 in the Southern part of the forest of Baïnem (longitude 2° 57′ 30″ and latitude 36° 47′ 35″) with a total of 86 trees, on a slope of 5%.

The *C*. *sempervirens* artificial stand (45 trees) was situated on the arboretum Bert trench in the Northern part of the Baïnem forest (longitude 2° 57′ 36″ and latitude 36° 47′ 49″). A natural *C*. *sempervirens* stand appeared in the southeast part of Baïnem forest after the disappearance of the bald cypress, *Taxodium disticum.* It consisted of 67 trees on a slope of 5 to 10 % with a Northwest exposure (longitude 2° 57′ 40″ and latitude 36° 48′ 2″). A hedge of *C. sempervirens* that separated the *Pinus* and Eucalyptus stands was also included in this study. They grew on a 5 to 10% slope exposed to the Southeast.

In March 1995 20 trees bearing seed cones, yet unpollinated, were selected throughout the stand of *C*. *sempervirens.* Three seed-cone-bearing branches were tagged on each tree. The position of the cones on each branche was mapped. This cohort of seeds cones was monitored periodically until seeds were mature, in June 1996. During this period the sample branches were examined eight times (three each in 1995 and 1996, and twice in 1997), at approximately four month intervals. During each visit, the possible cause of injury or death was recorded. A sample of the damaged cones was collected at each visit and dissected to confirm the cause of mortality.

### Species richness and damage assessment

Standardized cone collections were carried out every 15 days between December 1994 and August 1996 in the 4 stands in the Baïnem Arboretum. In each stand, two 40 cm-branches (one from the lower crown and another from the upper-crown) were selected randomly from each of 10 trees. First, the branches were beaten immediately over a net to collect the insects present on the cone surface as well as from the surrounding foliage. All the 1-year-old cones (i.e., green cones that are in the growth process), 2-year-old cones (i.e., ash-grey cones that completed the growth period the year before, and that will release seeds the following winter), and 3-year-old cones (i.e., overmature cone) were counted, and removed from the branch. Characteristics of the stand (climate, exposure) and of the sampled trees (i.e., type of crown: horizontal *vs.* pyramidal) and tree position in the stand were also noted at the time of sampling

In the laboratory, the 1-year-old cones and half of the 2 and 3- year-old cones were dissected to look for internal insect damage and for the presence of larvae. For each dissected cone, the seeds were extracted, and each seed lot was individually irradiated with X-rays using a Faxitron-43855® apparatus (20 Kv, 3 mA, 4 min) and recorded using X-ray sensitive film (Kodak® “Industrex M”). The seed quality (i.e., insect-infected seeds, larval development) was assessed from the radiographic images.

### Emergence period and sex-ratio of *M. wachtli*

The remainder of the 2 and 3-year-old cones were placed into rearing boxes (50 × 35) and stored in an outdoor insectary at Baïnem, Algiers. Adult insects were killed at emergence and identified to species. The emergence pattern of both sexes of the *Megastigmus* species was monitored daily during the 1998 autumnal period from 140, 2-year-old cones seed of *C*. *sempervirens* collected in natural stands before any emergence of the adults to investigate possible differences in the emergence period among sexes of the infected seeds. The sex ratio of the *M. wachtli* adults that emerged in September 1998 from seeds collected from *C*. *sempervirens* collected in natural stands during the summer of the same year was measured.

### *M*. *wachtli* development

To check if the female *M. wachtli* can develop parthenogenetically, 48 unfertilized females that emerged in September and October 1995 from individual infested seeds held in the laboratory, were fed honey water, and then placed into tulle bags that were attached to a branch containing cones in 4 trees, 2 bags per tree. Six females were put in each bag to observe if they produced progeny, and their gender. For comparison, 36 mating pairs of *M. wachtli* were introduced into 12 bags placed in 6 trees (2 bags per tree). Three pairs were put per trap to observe the emergence of progeny the following year.

Longevity of adult *M*. *wachtli* was determined under laboratory conditions and corroborated with periodic field observations. In the field, longevity of individuals was studied by following 904 adults emerged from seed of 2-year-old *C*. *sempervirens* cones in September 1995 and placed on the day of emergence in groups of identical emergence date into rearing boxes stored in an outdoor insectary at Baïnem and fed honey water. In a laboratory experiment, longevity of 279 adults of *M. wachtli* was studied during April and May 1996. The adults emerged from seed from 2-year-old *C*. *sempervirens* cones.

### Egg development in *M*. *wachtli* and *P*. *tessulatana*

The development of eggs was examined by dissection of ovaries of thirty *M*. *wachtli* females that emerged in September 1998 from seeds of 2-year-old cones of *C*. *sempervirens.* These females were placed on the first day of emergence in groups of identical emergence date into rearing boxes stored in an outdoor insectary at Baïnem and fed honey water. The chalcids were dissected by removing the complete ovary. The ovarioles were transferred to a slide, placed in Bouins fixative, and the eggs were counted. Microscopic observations were made on the oocytes of thirty females 1 to 15 days old. Development of the oocytes was recorded as pre-vitellogenic and vitellogenic oocytes, or fully developed eggs.

Egg development in *P. tessulatana* was determined as described above for *M. wachtli.* Microscopic observations of were made on the ovaries of twenty females 1 to 16 days old.

### Relationship between *M. wachtli* and *P. tessulatana*

The relationship between colonization by a cypress seed chalcid and the tortricid was characterized by their presence/absence ratio in 1000 colonized 2-year-old cones of *C*. *sempervirens.* 10 cones were collected from each of 100 trees. Cones attacked by *M*. *wachtli* and *P. tessulatana* were identified on the basis of their external appearance, as they dried and opened precociously when compared to cones containing healthy seed. For each attacked cone, the presence or the absence of attack of the chalcid and the tortricid was noted to see whether *M. wachtli* prefers, rejects or is indifferent to the previous attack of the cones by *P. tessulatana.*

### Distribution of the emergence holes of *M*. wachtli and *P*. tessulatana

To determine the relative distribution of *M*. *wachtli* and *P. tessulatana* damage to the cones of *C*. *sempervirens* and *C*. *arizonica*, external damage and the position of the adult's emergence holes on the cones was noted and drawn. 244 cones infested by *M. wachtli* and 62 cones infested by *P. tessulatana* were collected from 80 *C*. *sempervirens* trees. Cones having weak and strong *M*. *wachtli* damage were examined separately. 30 cones infested by *M. wachtli* and 60 by *P. tessulatana* were collected from 50 *C*. *arizonica* trees and examined.

### Damage by *Caruslaspis minima* Targioni (Homoptera, Diaspididae), to seed cones of *C*. *arizonica glabra*

In December 1994, at *C*. *arizonica glabra* stand, 2 branches bearing 1 and 2-year-old cones were randomly chosen from each of 10 flowering trees selected randomly. A total of 62 sound cones and 20 infected-cones by *C*. *striata* were collected. At the laboratory, the 1 and 2-year-old cones were dissected and the seeds were extracted. All seed lots were individually irradiated with X-rays to evaluate the respective number of normal, *C*. *minima*-infested, and empty seeds per cone.

### Insect damage to cones of *Cupressus dupreziana*

513 cones were selected from 2-and 3- year-old cones of *C. dupreziana* that had been collected in April 1995. 496 cones were selected from 2-and 3-year-old cones collected in June 1995 in Tassili. Thirty surviving cones, those in their third year of development, were harvested just before the natural seed dispersal occurred and the seeds were removed. A total of 358 seeds were individually irradiated with X-rays to evaluate the respective number of filled, insects-damaged and empty seeds per cone.

### Data treatment

The percentage of cones colonized by *Megastigmus* were compared among tree, stand, and species using analysis of variance (Anova, Statsoft Statistica/W package). ANOVA was used to compare the percentage of *M*. *wachtli* and *P. tessulatana* emergence holes on the colonized cones, and to test localization of the colonization per cone shape, insect pests and cypress species. The percentages data were transformed by arcsin √ p to achieve homogeneity of the variance before statistical analysis. ANOVA was followed by Tukey's test to look for differences between locations and species. The results of the longevity of *M. wachtli* were analyzed by the χ^2^ test. The relationship between the colonization by the seed chalcid and by the tortricids was subjected to a Pearson correlation test (R).

## Results

### Entomofauna and description of insect damage on cypress seed cones

Seven insect species and one diaspine cochineal were observed to attack the cones and seeds of *Cupressus* in the Baïnem arboretum during 1995 and 1996. According to the feeding habits defined by Turgeon et al. (1994), two species are conophagous (i.e., feed on cone tissues only), one is conospermatophagous (i.e., feed on both cone tissues and seeds), and three species are spermatophagous (i.e., feed on seeds only), the diaspine cochineal is opophagous (i.e., suck the sap contents of the scales) (Table 1). The three cypress species studied, *C. arizonica arizonica*, *C. arizonica glabra* and *C. sempervirens*, were attacked by four main pests: *O. maculatus*, *O. depressus*, *M*. *wachtli* and *P. tessulatana.* Two other species, *Brachyacma oxycedrella* and *Nanodiscus transversus* were also found on *C*. *sempervirens.*

Two of these species, the spermatophagous *M*. *wachtli* and the conospermatophagous *P. tessulatana*, had already been observed on the cone and seeds of *C*. *sempervirens* and *C*. *macrocarpa* in the Meurdja arboretum and Chrea National Park (Bouaziz 1993; Bouaziz and Chakali 1997). We found during two years of observation that *M*. *wachtli* emerged from mature cones and laid eggs in seeds of green fully-grown cones that had just begun the seed maturation phase. The conospermatophagous *P. tessulatana* presented two generations per year with a third partial generation. The same observation was made by Guido et al. (1995) in Italy.

The conospermatophagous *P. tessulatana* attacked green cones during the first year of development. Three spermatophagous pests, *M*. *wachtli*, *O. maculatus* and *O. depressus* attacked maturing and mature seeds inside 2-and 3-year-old cones (Table 1). The phenological relationships between cone development and pest attacks from bud burst to seed dispersal were similar to those reported by Guido et al. 1995.

Another species, *Caruslaspis minima* Targioni (Homoptera, Diaspididae), was abundant on the cypress cones studied. These insects were unusual. They were motionless and live in colonies, fixed on the various aerial parts (sheets, fruits, branches and trunk). They show an important sexual dimorphism. The females which were, in general, the most abundant forms and most recognizable in nature, were protected by a shield. This diaspine cochineal caused yellowing of the attacked parts where sap was sucked from the plant, but otherwise did not impact the seeds.

**Table 1.**
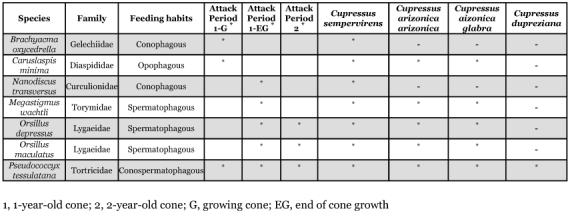
List of pest insects of the cypress cones indexed in the Baïnem arboretum.

The total number of seeds in sound cones of *C*. *arizonica glabra* was 119.450 ± 76.762 (Mean ± SD) (n = 62) and the total number of seeds in cones infested by *C*. *minima* was 135.452 ± 96.982 (n = 20). Analysis of the x-ray radiographic image of the overall seed yield of *C*. *arizonica glabra* cones collected at maturity revealed that an average percentage of 77% of the seeds were filled in sound cones, and 23 % of the seeds were empty and aborted. X-ray analyses of the seed content of *C*. *minima* cones showed that 89.4 % of seed were filled, and 10.6% of the seeds were empty and aborted. The percentages of normal, aborted and empty seeds per cone were not significantly different among sound and damaged *C*. *minima* cones (ANOVA: F 1.80 = 2.701, P = 0.104).

Symptoms of insect attack were observed on the cones of *C*. *dupreziana.* The percentage of infestation of 2-year-old cones varied from 46.77% to 52.24%. However, it was not possible to identify the insects that were responsible for this damage. The cones were attacked by Lepidoptera whose exit holes shapes were irregular and of a size identical to those of *P*. *tessulatana.* The X-ray analyses of seeds extracted from 30 attacked cones showed that only 2.5% of the seeds were normal. *C. dupreziana* did not manage to ensure its regeneration. The attack of the pest seed cones, presumably *P. tessulatana*, seemed to be one of the causes.

### Other factors causing mortality

Once seed cone buds have been formed, their development can be stopped by several factors such as climatic conditions that can cause abortion either of buds before they burst open, or of immature seed cones. In most cases, abortion of seed cones was the result of poor or inadequate pollination.

Tagged cones were attacked by several fungi, the most important species of which were *Seiridium* (=*Coryneum*) *cardinale* (Wag.), also responsible for disease known as cypress canker, and *Pestalotia* (=*Pestalotiopsis*) *funerea* Desm. The fungus attack was either limited to cone scales or it extended to the whole cone. The fungi could easily penetrate the seed coat, on which they produced abundant spores (Bouaziz 2003).

### Biology of *M*. *wachtli*

**Figure 1.**
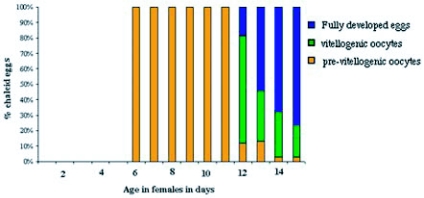
Percentage of eggs at different stages in the ovaries of female cypress seed chalcids, *Megastigmus wachtli*, from thirty females 1 to 15 days old, collected in September of 1995.

The female reproductive system of *M*. *wachtli* was composed of two ovaries, each with six ovarioles. At emergence, the ovarioles were not differentiated. The female abdominal content was essentially fatty tissue that regressed after the second day of feeding. The genital apparatus did not differentiate until after the sixth day of feeding when the genital apparatus formed. From the 12th day of feeding, the presence of one egg was observed in vitellogenesis. It thus seemed that this species could start to lay eggs only after the twelfth day (Figure 1). The average fecundity of a female under our rearing conditions was 7.8 ± 6.4 eggs (n = 20) (±SD). This observation differed from that made on other *Megastigmus* species, such as *M*. *spermotrophus*, where the females were able to lay eggs shortly after emergence (Hussey 1955; Roux 1995).

When virgin females were placed in bags in the absence of males they produced 45 males and 0 females in October 1995. In 1996 the fertilized females of the same origin laid eggs producing 38 females and 37 males (Table 2). These results support the evidence that *M*. *wachtli* is capable of arrhenotokous parthenogenesis as are other species of *Megastigmus* (Roux 1995).

**Figure 2.**
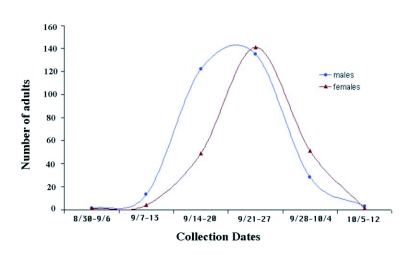
Emergence of males and females of *Megastigmus wachtli* from seeds of *Cupressus sempervirens* collected in autumn of 1998 (number of collected cones = 140) in a natural stand of Baïnem. Drawn according to Camus (1914).

**Figure 3.**
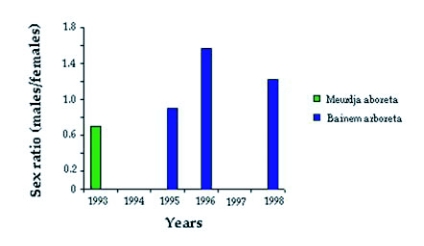
Sex-ratio of the *Megastigmus wachtli* adults that emerged during the summer 1993 from seeds collected during the spring of that year and the autumn of 1995. Data for 1996 and 1998 were from seeds collected during the summer of these years.

In stands surveyed both years, the most abundant sex appeared to vary among years, with males becoming most abundant in Baïnem arboretum in 1996 and 1998 (Figure 3). The ratio of males to females was 1.6 in 1996 (n = 408) and 1.2 (n = 549) in 1998. The situation was completely different in 1993 and 1995 when the sex ratio was 0.7 in favor of females in 1993 in the Meurdja arboretum (n = 114) (Bouaziz 1993; Bouaziz and Chakali 1998) and 0.9 in 1995 (n = 1531). This sex ratio suggested that M. wachtli was capable of arrhenotokous parthenogenesis as are other species of Megastigmus. The same result was shown by Roques et al., (1998) for *M. wachtli* in natural and introduced trees in Greece where they obtained a sex ratio in favor of females in 14 sites, 5 sites in favor of the males, and 4 sites had a sex ratio of 1.1. Ben Jamaa and Roques (1997) found an average sex ratio of 0.8 in favor of the females.

Radiography of the seeds allowed the examination of later development of larvae during cone maturation. Larval development proceeded entirely in a single seed. The larva initially developed at the expense of the cotyledons and it started to consume seed endosperm the following spring. Similar results were observed previously (Roques 1983; Roques et Raimbault 1995; Guido et al. 1995). Larvae of *M*. *wachtli* seemed able to develop in non-fertilized ovules as Guido et al. (1995) suggested, but we could not experimentally infest non-fertilized ovules in order to test this assumption (Bouaziz and Roques, unpublished results). Rouault et al. (2004) hypothesize that all species of Megastigmus associated with Pinaceae can oviposit in unfertilized ovules, whereas those exploiting Cupressaceae cannot, and thus oviposit only in already fully developed fertilized seeds. Infested megagametophytes of unpollinated ovules did not degenerate as would have been expected, but continued to develop (Aderkas et al., 2005). Niwa and Overhulser (1992) and Rappaport et al. (1993) showed that the *M*. *spermotrophus* larvae were able to lay eggs on non-fertilized ovules of the *Pseudotsuga* species and that the larva could complete its development there. The chalcid lays its eggs in seeds of Douglas fir (*Pseudotsuga menziesii* (Mirbel) Franco) before fertilization has taken place in the plant. Oviposition not only prevents the expected degeneration and death of unfertilized ovules, but it induces energy reserve accumulation (Aderkas et al., 2005). Following oviposition by *M*. *spermotrophus* in unpollinated megagametophytes, larvae hatched and began consuming the central zone. Eggs were laid in megagametophytes that were differentiating archegonia which would eventually house the plant eggs. (Aderkas et al., 2005). The torymid chalcid wasp was able to induce identical nuclear behavior in infested, unfertilized megagametophytes, as occurred in uninfested, fertilized megagametophytes (Aderkas et al., 2005).

**Table 2.**
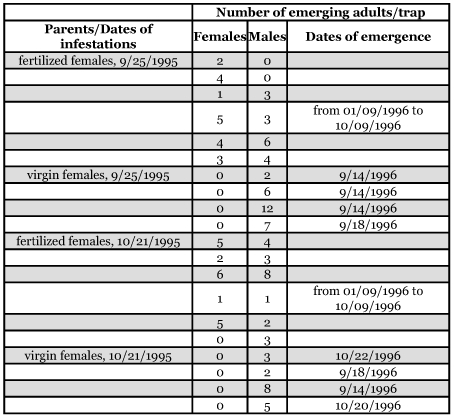
Resulting emergence of males and females of the cypress seed chalcid, *Megastigmus wachtli*, from eggs laid in seeds of *Cupressus sempervirens* by unfertilized female *M. wachtli.*

The emergence of *M*. *wachtli* was spread out over 6 weeks from the beginning of September to mid-October (Figure 2). Emergence of males was earlier as 50% of males emerged before the 21^st^ of September compared to the 23^rd^ of September for 50% of females. These emergences were later than those observed in the natural sites in Greece and in France but earlier than those of Tunisia (Roques et al. 1998).

**Figure 4.**
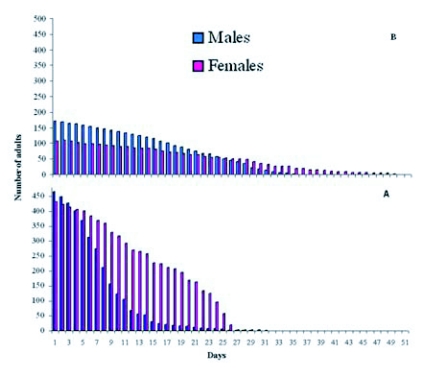
Adults longevity of a cypress seed chalcid, *Megastigmus wachtli*, emerged from seed yield of 2-year-old cones *Cupressus sempervirens* in September (a) in field, placed into rearing boxes stored in an outdoor insectary at Baïnem and nourished with honey water; (b) breeding under laboratory conditions.

The females had an average lifespan significantly longer than that of males. 13.35% of males died at the end of 8 days and only 4.87% of them survived 15 days or more. In contrast the corresponding percentages for females were 2.31% died at the end of 8 days and 7.18% were still alive after 15 days, many of which survived up to 27 days. Given that eggs did not start to mature until day 12, these results suggested that the only these older females would lay eggs. In the field, preliminary observations showed that the lifespan could increase up to 32 days (Figure 4). Breeding in the laboratory showed that longevity could reach 50 days (Fig. 4). This suggests that emergence can occur over 6 weeks and the insects can live up to 32 days under natural conditions.

**Table 3.**
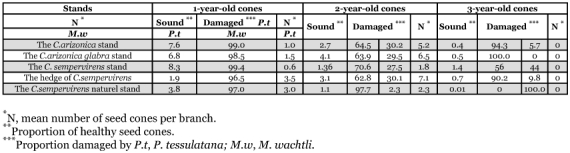
Cone crop abundance and pest damage to cypress seed cones of different ages collected between 1994 and 1996 in the Baïnem arboretum.

### Insect damage to cones

Pest damage varied with stand, cypress species, and stages of cone development (Table 3). Damage resulted mostly from feeding by *M*. *wachtli* which dominated the pest complex in 5 of the stands, and destroyed up to 30% of 2-year-old cones and 44% of 3-year-old cones of *C*. *sempervirens*, 30% of 2-year-old cones of *C*. *arizonica*, and 29.5% of 2-year-old cones of *C*. *glabra.* In most of the stands, the average percentage of trees that were attacked by *M*. *wachtli* varied significantly. Some trees were free of heavy attacks whereas others had more than 80 % of their cones colonized as observed for the *C*. *arizonica* stand (F34, 228 = 6.057, P = 0.000), the artificial parcel of *C*. *sempervirens* (F22, 12 = 10.05, P = 0.000) and the hedge (F25, 15 = 9.92, P = 0.000). On the other hand, no significant difference in tree attack by chalcids was observed on trees of the *C*. *sempervirens* natural stand.

**Table 4.**
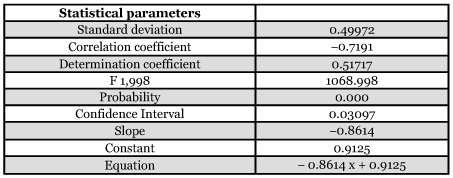
Regression analysis of cones attack by *M. wachtli* and *P. tessulatana*

Differences in damage caused by *M*. *wachtli* were also noted between sites and species. The *C*. *sempervirens* artificial stand (40.7%), and the *C*. *arizonica glabra* stand (28%), were significantly more infested than the *C. arizonica* stand, and the *C*. *sempervirens* natural stand and hedge (F 4, 131 = 4.02, P = 0.004). Tukey's test showed that the infestations in the *C*. *arizonica* stand (23%) and the *C*. *sempervirens* natural stand (2.8%) and hedge (21%) were not significantly different. These differences in infestation corresponded to insect population fluctuations (Da Ros et al. 1993), or to variation in cone production between sites (Roques 1983).

Conophagous pests infested only 3.5 % of the cones of *C*. *sempervirens* during the first year of development. These losses were due mainly to the first generation of *P. tessulatana.* Guido et al. 1997 reported similar results in Italy, where *P. tessulatana* infested only 10% of the cones. In natural and naturalized stands in Greece, damage resulted mostly from feeding by *P. tessulatana* larvae which dominated the pest complex in 13 of the stands, and destroyed up 98% of 1-year-old cones in Eleousa (Rhodos) (Roques et al. 1997). Attack by larvae of the first generation of the tortricid stopped the growth of seed cones; the cones dried up prematurely and usually dropped to the ground. Only the slightly attacked or healthy cones then remained on the tree. The limited decrease in the number of 2-year-old cones resulted mainly from feeding by *P. tessulatana* larvae of the second and third generation. Unlike what happened to 1-year-old cones, most of the damaged cones did not disappear from the branch, and seeds that were not damaged directly were able to reach maturity. However, the apparent limited impact of insect attack during the cone maturation phase is misleading because damage by spermatophagous insects such as *Orsillus* spp, which can be detected only by X-ray analysis, was not taken into consideration. No significant difference in attack by tortricids was observed between sites and species (F 4, 131 = 0.885, P = 0.475).

**Figure 5.**
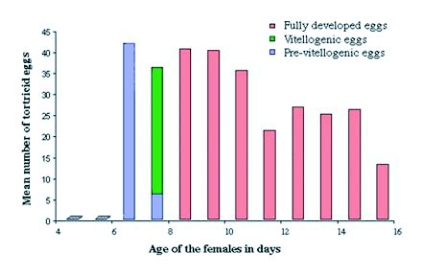
Mean number of eggs at different stages in the reproductive apparatus female of cypress tortricids, *Pseudococcyx tessulatana*, on twenty females from of 1 to 16 days old, collected in April, 1995.

### Reproduction by *P. tessulatana*

**Figure 6.**
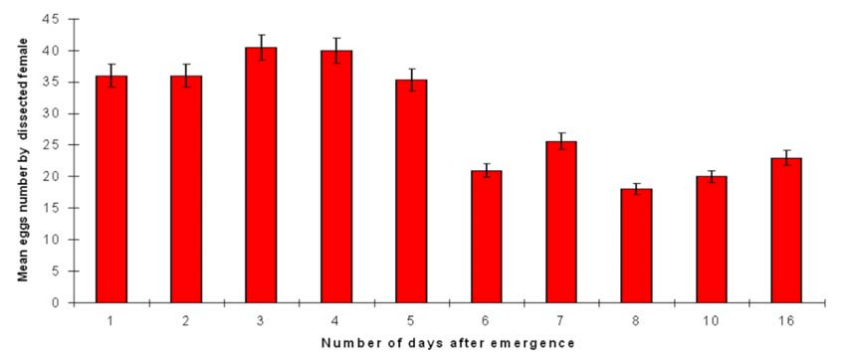
Mean eggs number by female of *Pseudococcyx tessulatana* dissected several days after emergence.

The female reproductive apparatus of *P*. *tessulatana* was composed of two ovaries, with four ovarioles in each. Previtellogenesis lasted less than two days. In general, mature eggs were present by the third day after emergence (Figure 5). Zocchi (1963) observed that *P. tessulatana* females laid eggs after mating. The length of the mature egg varied from 0.9 to 1 mm. These eggs were attached to the surface either isolated or in series. From the sixth day, egg laying seemed to have begun as the dissections showed that the ovarioles contained few eggs. Eggs can be laid for over 16 days (Fig. 6).

**Figure 7.**
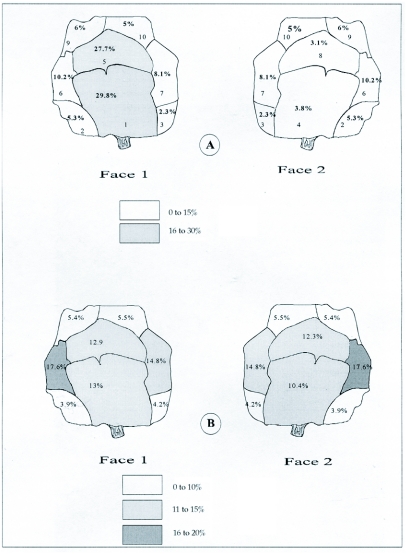
Distribution of the emergence holes of *Megastigmus wachtli* according to the degree of damage of the cones of *Cupressus sempervirens.* A: 1 to 9 emergence holes by cone. B: more than 10 emergence holes by cone. Drawn according to Camus (1914).

**Figure 8.**
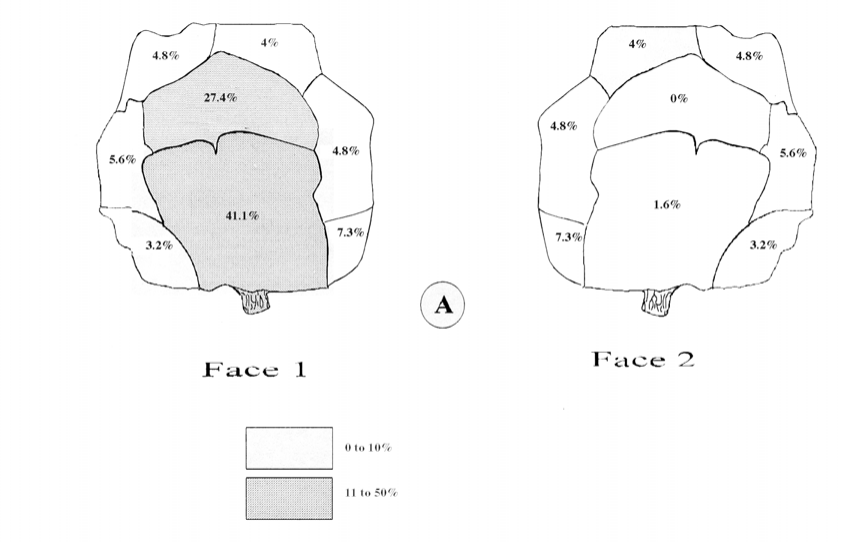
Distribution of the emergence holes of *Pseudococcyx tessulatana* of the cones of *Cupressus sempervirens.*

### Relationships between the seed chalcid and tortricid

The Pearson (R) correlation coefficient of sampling, which reflects the degree of linearity between the attacks of the two insects on the same cone, was negative (R = -0.72). The attacks of these two pests seemed to exclude each other (Table 2). In the event of a strong cone production, *M. wachtli* chose the cones not colonized by *P. tessulatana.*

**Table 5.**
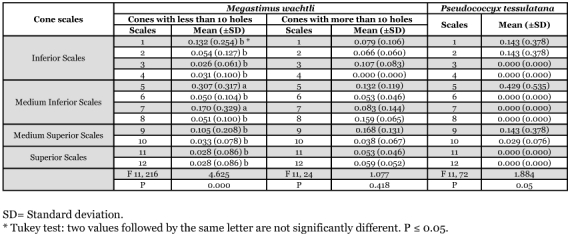
Variance analysis of *M*. *wachtli* and *P. tessulatana* emergence holes repartition on the *C. sempervirens* lengthened shaped cones.

### Distribution of the emergence holes of *M. wachtli* and *P. tessulatana*

The distribution of the emergence holes of *M. wachtli* on the colonized cones is illustrated in Figure 7. This distribution reflects the distribution of the insects inside the cone since larvae are unable to move to different parts of a cone.

In the event of low level damage, the majority of the emergence holes were found together on the level of two cone scales, the lowest and the medium ones situated only on one face of the cone number of holes = 2047, F9 2037 = 45.648, P = 0.000). The highest cone scales had a limited number of emergences holes. Thus, the damage was concentrated certain regions.

**Table 6.**
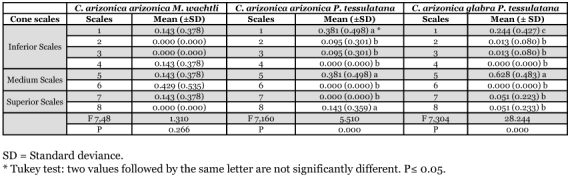
Variance analysis of *M*. *wachtli* and *P. Tessulatana* emergence holes repartition on the cones of *C*. *arizonica arizonica* and *C. arizonica glabra.*

In the event of heavy damage, *M*. *wachtli* emergence holes were observed on both faces of the cone. They were distributed on the two lowest scales and the medium scales, among which one was significantly more colonized (number of holes = 350, F9, 340 = 4.780, P = 0.000). The two sides of the lowest scales and the highest scales were significantly less colonized. This could mean that M. wachtli preferred to lay eggs on seeds that were near one another.

The long-shaped cones were attacked differently than the round cones. In the event of low-level attack, the majority of the attacks on long cones were located on two scales, one front lower medium (30.7%) and one on the side lower medium scales (17%) (number of holes = 226, F 11,216 = 4.625; P = 0.000) (Table 5). The preference areas of exit holes probably corresponded to the laying place also located on only one face of the cone. As discussed above, the attacks were concentrated in space. The lower and higher scales were significantly more attacked. In the event of heavy attack, no significant differences were observed in distribution of the holes of exit of *M*. *wachtli* between the scales. The insects thus randomly attacked the various scales of the cone contrary to what was observed in the round cones.

Attack by *M. wachtli* on *C. arizonica* cone scales was random (number of holes = 58, F 7, 48 = 1.310, P < 0.266) (Table 6). Attack by *P. tessulatana* on *C*. *sempervirens* was concentrated on the lower and medium scales located on the same face of the cone (number of holes = 620, F 9, 610 = 15.197, P = 0.000) (Figure 8). In comparison, attack on *C. arizonica* was condensed on three scales: one lower, one medium and one higher located on the same face of the cone (number of holes = 170, F 7, 160 = 5.51, P = 0.000) (Table 6). The majority of the damage to the *C*. *arizonica glabra* variety was centralized on two scales, a lower and a medium. The medium cone scale was significantly more colonized than the lower one (number of holes = 40, F 7, 30 = 28.24, P = 0.00). Thus, attack strategy by both the chalcid and tortricid varied according to the cypress species and location of attack on the cone.

## Discussion

The study of *M*. *wachtli* biology showed that the female was not different from other species of *Megastigmus.* The emergence of *M*. *wachtli* from seed cones usually lasted 42 days, and adults lived for about 30 days, but only the older females lay eggs. Therefore, cones must be protected from infestation during a period of 9 weeks.

The *M. wachtli* laying strategy on the circumference of the medium scales was probably advantageous for the discovery of full seeds concentrated at this level. The attack was different on the long vs. round cones; the lower scales were significantly less attacked in the event of low-level infestation of long cones. In comparison, the strategy of attack of *P. tessulatana* was different on the two varieties of *C*. *arizonica,* and was different from its attack on *C*. *sempervirens.* Oviposition sites for *M. wachtli* and *P. tessulatana* were similar, as the latter probably chose the laying sites of *M*. *wachtli* for ovipositing its eggs. The antagonism of the attacks of these two species can be explained by the choice of the same favorite area for attack.

The competition between these species for use of cypress cones suggests that they use different strategies for different species of cypress. The number of insects that could develop relative to the number of cones available also varies between species of cypress. *M. wachtli* has a high potential for colonization because it has evolutionary advantages that enabled it maintain its populations including its capacity for parthenogenesis, its fecundity and longevity.

During our study, *O.depressus* was observed on the cones of *C. arizonica arizonica*, *C*. *arizonica glabra* and *C. sempervirens.* It seems that it is more widespread in warm areas because it was found less in the northern Mediterranean where *O. maculatus* has replaced it, except in Portugal (Battisti, personal communication). Indeed, in Portugal, it was shown that *O.depressus* affects the production of seeds in the plantations of cypress (Ramos and Abrantes 2000).

Unlike in southern Europe, the seed bug *O. depressus* seems more abundant than *O. maculatus* on cypress species growing in North Africa (Bouaziz and Chakali 1997). In Europe and in the Mediterranean basin, trees of the Cupressaceae family host several seed bug species of the genus *Orsillus*, especially O. *maculatus* and O. *depressus.* Detailed information about natural history is available for O. *maculatus* (Guido et al 1995; Battisti et al 1997; Ramos and Abrantes 2000). This seed bug develops essentially on the evergreen cypress, *C. sempervirens*, where it lays eggs in cone openings, such as emergence holes of a cypress seed chalcid, *M. wachtli*, or at inner side of partly detached cone scales (Battisti et al 2000). Both nymphs and adults feed on mature seeds inside the cones, causing considerable damage to seed crops in seed orchards as well as in natural stands (Roques et al. 1999; Battisti et al 2000). Information on the biology of *O. depressus* is much less detailed but this bug has been observed on several species of *Juniperus*, *Chamaecyparis* and *Thuja* as well as on *Cupressus* ssp and *Pinus* (Cleu 1953; Dupuis 1965; Dioli 1991; Stichel 1962). In addition, it was reported to severely affect seed production on a plantation of *Cupressus lusitanica* Mill. in Portugal (Ramos and Abrantes 2000). The seed bug *O. maculatus* is associted with pathogenic fungi affecting cypress, such as *S. cardinal* which is responsible for the cypress bark canker, and *Pestalotiopsis funerea* (Desm.) The adult bugs may disseminate the fungi conidia among trees whereas the nymphs may find a suitable development site in fungus-infested cones (Battisti et al 1999; Ramos and Abrantes 2000). In Algeria, a fungus-infected cone can be inhabited by the nymphs of the seed bug *O.depressus*, the adults of which may carry a heavy spore load at emergence. Cones are infected when eggs are laid within the cone, most frequently via the emergence holes of *M. wachtli* (Bouaziz 2003).

*Brachyacma oxycedrella* (Millière) (Lepidoptera: Gelechiidae) was observed on the cones of *C*. *sempervirens* during August 1996. This species was observed in all southernmost Europe, from Spain to Damaltia, on *J. oxycedrus*, *J. thurifera*, *J. phoenicea*, *Biota orientalis* and *C*. *sempervirens* and in Algeria on *T. articulata* (Roques 1983). Guido et al. 1995 also observed this species on *C*. *sempervirens* in Italy. It is widespread in Morocco, where it was observed along the Atlantic littoral (Northern) and in the continental plains (Marrakech) on *C*. *sempervirens* as well as in high Atlas mountains on *J*. *thurifera* and *J. oxycedrus*, in the middle Atlas mountains and on the Eastern plateau (Oujda) on *J. thurifera* (El Hassani and Messaoudi 1986; El Alaoui El Fels 1997).

*Nanodiscus transversus* Aubé (Coleoptera: Nanophyidae) was also observed on several species of Juniperus in the Meurdja arboretum (Bouaziz 1993; Bouaziz and Chakali 1997). Adults were observed during the first 10 days of March 1996 on the cones of *C*. *sempervirens. N. transversus* is widespread in the south of France, Spain, Sicily, Greece, Italy and Algeria on *Juiperus oxycedrus* and *J*. *phoenicea*, and was observed occasionally on the cone of *C*. *sempervirens* in France (Roques *et al* 1984). In Morocco, it attacks *Tetraclinis articulata* and *J*. *oxycedrus* (El Hassani and Messaoudi 1986).

Despite the sampling problems Algerian fauna presented a notable difference from those already known in Italy and France. The acarina *Trisetacus juniperinus* that is known in the whole Mediterranean and is a very important pest, where its attack is responsible for the disappearance of one-year-old cones, was not found in this study. However, similar damage (hypertrophied seeds, drying of the first year cones, premature fall of cones) was observed on several occasions on the *C*. *sempervirens* cones in the Baïnem arboretum. That suggests the probable presence of this species on the *C*. *sempervirens* cones in Algeria. Another species of minor importance, *Ernobius cupressi* that was frequent in Kos was not inventoried in this study. However, Algerian fauna appears more diverse than in Morocco.

## References

[bibr01] Aderkas P Rouault G Wagner R Chiwocha S Roques A 2005 Multinucleate storage cells in Douglas-fir (*Pseudotsuga menziesii* (Mirbel) Franco) and the effect of seed parasitism by the chalcid *Megastigmus spermotrophus* Wachtl. *Heredity* 94 616 622 15829985 10.1038/sj.hdy.6800670

[bibr02] Aderkas P Rouault G Wagner R Rohr R Roques A 2005 Seed parasitism redirects ovule development in Douglas fir. *Proceedings of the Royal Society-B* 272 1491 1496 16011924 10.1098/rspb.2005.3061 PMC1559829

[bibr03] Battisti A Cantini R Feci E Frigimelica G Guido M Roques A 2000 Detection and evaluation of seed damage of cypress, *Cupressus sempervirens* L. in Italy. *Seed Science and Technology* 28 199 208

[bibr04] Battisti A Cantini R Rouanlt G Roques A 2003 Serotinous cones of *cupressus sempervirens* provide viable seeds in spite of high seed predation. *Annals of Forest Sciences* 60 781 787

[bibr05] Battisti A Colombari F Frigimelica G Guido M 1997 Life history of *Orsillus maculatus*, damaging seeds of *Cupressus sempervirens.* *Proceedings of the 5th Cone and Seed Insects Working Conference*, September 1996 215 220 Padova, Italy Institute of agricultural entomology

[bibr06] Battisti A Colombari F Frigimelica G Guido M Roques A 1999 Efficient transmission of an introduced pathogen via an ancient insect-fungus association. *Naturwissenschaften* 86 479 483 10541657 10.1007/s001140050658

[bibr07] ben Jamaa ML Roques A 1997 Survey of impact on seed cones of two species of Cupressaceae, *Cupressus sempervirens* L. and *Tetraclinis articulata* Mast. in Tunisia. Proceedings of the 6th Arabian Congress for vegetal protection Beyrouth October 1997

[bibr08] Bouaziz K 1993 *Contribution à l'étude des insectes des cônes dans l'arboretum de Meurdja et dans la Cedraie de Chrèa*. Thèse d'Ingénieur Agronome Institut National Agronomique, Alger 80

[bibr09] Bouaziz K 2003 *Etude d'un modèle de relation tripartites cône-insecte-champignon: rôle de la punaise Orsillus depressus Dallas (Heteroptera : Lygaeidae) dans la vection du champignon pathogène d'origine exotique*, *Seiridium cardinale (Wag.) Sutton and Gibson*, *responsable de la maladie du chancre du cyprès en Algérie*. Thèse Docteur d'Université d'Orléans France 210

[bibr10] Bouaziz K Chakali G 1997 Diversity and impact of cone and seed insects in Algeria. In: Battisti A, Turgeon JJ, editors. 1997. Proceedings of the 5th Cone and Seed Insects Working Party, Page 193 – 207. Conference (UFRO S7.03.01), September 1996, Monte Bondone, Italy. Padova Institute of Agricultural Entomology, University of Patova

[bibr11] Camus A 1914 *Les cyprès (genre cupressus) Monographie*, *Systematique*, *Anatomie*, *Culture des principaux usages*. Paris Paul Lechevalier

[bibr12] Cleu H 1953 Biogéographie et peuplement entomologique du bassin de l'Ardéche. *Annals of Society of Entomology in France* 122 1 74

[bibr13] Da Ros N Ostermeyer R Roques A Raimbault JP 1993 Insect damage to cones of exotic conifer species introduced in arboreta I. Interspecific variations within the genus *Picea*. *Journal of Applied Entomology* 115 113 133

[bibr14] Dioli P 1991 Presenza di *Orsillus dupressus* Dallas, 1852 nella zona alpina osservazioni sulle specie italiana del genere (Insecta, Heteroptera, Lygaeidae). *Il**Naturalista Valtellinese*, *Atti del Museo Civico di Storia Naturale di Morbegno* 2 47 51

[bibr15] Dupuis C 1965 Etude de L'oligophagie de trois punaises des genévriers. *Cahiers des Naturalistes*, *Bulletin N*. P 2 105 122

[bibr16] El Alaoui El Fels MA 1997 Entomofauna of some conifers in the occidental High Atlas mountains (Morocco). Battisti A Turgeon J Proceedings of 5th cone and seed insects working party, Conference, Monte Bondone, Italy. Institute of Agricultural Entomology University of Patova 21 26

[bibr17] El Hassani A Messaoudi J 1986 The cone and seed pests of conifers and their distribution in Morocco. Roques A Proceedings of the 2nd Conference of the Cone and Seed Insects Working Party 5 14 S2.07-01, Briancon, 05100 France September 3–5 1986

[bibr18] Guido M Battisti A Roques A 1995 A contribution to the study of cone and seed pests of the evergreen cypress *(Cupressus sempervirens* L.) in Italy. *Redia* 78 211 227

[bibr19] Guido M Battisti Roques A 1997 Mortality factors affecting cones and seeds insects of *Cupressus sempervirens* prior to seed dispersal. Battisti A Turgeon JJ Proceeding of 5th Cone and Seed Insects Working Party Conference, pp 209 – 214 (UFRO S7.03.01). September 1996, Monte Bondone, Italy. Padova Institute of Agricultural Entomology, University of Padova

[bibr20] Guido M Roques A 1996 Impact of the phytophagous Insect and Mite Complex associated With cone of Junipers (*Juniperus phoenicea* L. and *J. cedrus* Webb. And Berth.) in the Canary Islands. *Ecologia Mediterrane* 22 1 10

[bibr21] Hussey NW 1955 The life histories of *Megastigmus spermotrophus* Wachtl (*Hymenoptera: Chalcidoidae*) and ITS principal parasite with descriptions of the developmental stages. *Transactions of the Royal Entomological Society of London* 106 133 151

[bibr22] Niwa CG Overhulser DL 1992 Oviposition and development of *Megastigmus spermotrophus* (*Hymenoptera: Torymidae*) in unfertilized seeds. *Journal of Economic Entomology* 85 23 28

[bibr23] Ramos P Abrantes C 2000 Transmissao de esporosde fungos do genero *Seiridium* por *Orsillus* spp. (Heteroptera: Lygaeidae) in Portugal. *III Encontro Nacional de Plantas Ornamentais*, *Auditorio do Instituto Politecnico de Viana do Castelo.* Association Portuguese de Horticultura

[bibr24] Rappaport N Mori S Roques A 1993 Estimating impact of a seed chalcid *Megastigmus spermotrophus* Wachtl (Hymenoptera: Torymidae) on Douglas - Fir seed production: The new paradigm. *Journal of Economic Entomology* 86 845 849

[bibr25] Rizzotti Vlach M 1994 Popolamenti ad Eterotteri della Valpolicella (Veneto, Regione Veronese). *Memorie della Società Entomologica Italiana* 73 59 152

[bibr26] Roques A 1983 *Les insectes ravageurs des cônes et graines de conifères en France.* Paris Institut National de Recherche Agronomique

[bibr27] Roques A Battisti A 1999 Ravageurs du Cyprès. Teissier du Cros E Ducrey M Barthélémy D Pichot C Giannini R Raddi P Roques A Sales Luis J Thibaut B *Le Cyprès. Guide pratique* 74 95 Florence Studio Leonardo

[bibr28] Roques A Carcreff E Rasplus JP 1998 *Cupressus sempervirens* vs Cypress seed chalcid, *Megastigmus wachtli:* Genetic and evolutionary relationships, IUFRO S7.01 *Symposium physiology and genetics of trees. Phytophage interactions*. Versailles Institut National de Recherche Agronomique, Versailles

[bibr29] Roques A Raimbault JP Goussard F 1984 La colonisation des cônes et galbules des genévriers méditerranéens par les insectes et acariens et son influence sur les possibilités de régénération naturelle des essences. *Ecologia Mediterranea* 10 147 169

[bibr30] Roques A Raimbault JP 1986 Cycle biologique et répartition de *Megastigmus wachtli* (Seitner) (*Hymenoptera*, *Torymidae*), Chalcidien ravageur des graines de cyprès dans le bassin Méditerranéen. *Institut National de Recherche Agronomique* 101 370 381

[bibr31] Roques A Markalas S Roux G 1997 Entomofauna of seed cônes of evergreen cypress, *Cupressus sempervirens*, in natural and naturalized stands of Greece. *Proceeding of 5th IUFRO WP cone and seed insects*, *Bondone Trento* 2 – 7 *September 1996. Monte Bondone*, *Italy* Padova *Institute of Agricultural Entomology*, *University of Padova*

[bibr32] Roques A Skrzypczynska M 2003 Seed-infesting chalcids of the genus *Megastigmus* Dalman, 1820 (Hymenoptera: Torymidae) native and introduced to the West Palearctic region: taxonomy, host specificity and distribution. *Journal of Natural History Volume* 37 127 238

[bibr33] Rouault G Turgeon J Candau JN Roques A Aderkas P 2004 Opposition strategies of conifer seed chalcids in relation to host phenology. *Naturwissenschaften* 91 472 480 15729760 10.1007/s00114-004-0554-4

[bibr34] Rouault G 2002 *Biologie et répartition des punaises du genre Orsillus* (*Heteroptera: Lygaeidae*) *associées aux Cupressaceae: une étude de l'impact d'un pathogène introduit sur les interactions cônes-insectes.* Thèse Docteur d'Université d'Orléans

[bibr35] Rouault G Roversi PF Cantini R Battisti A Bouaziz K Roques A 2000 First record of an egg parasitoid (*Telenomus* GR. *Floridanus*, Hymenoptera Scelionidae) of two true seed bugs (Heteroptera Lygaeidae) living on Cupressaceae. *Redia* 83 163 173

[bibr36] Roux G 1995 *Etude comparative des mécanismes d'induction de la diapause prolongée chez quelques insectes spécifiques des cônes de conifères.* Thèse d'Université d'Orléans

[bibr37] Stichel W 1962 *Illustrierte Bestimmungstabellen der Wanzen II Europa (Hemiptera-Heteroptera-Europae).* 774 Berlin Hermsdorf

[bibr38] Zocchi R 1963 Insetti del Cipresso III. Note morfologiche - etologiche Sulla *Pseudococcyx tessulatana* Stgr. *(Lepidoptera*, *Tortricidae*). *Redia* 48 239 264

